# An individual-level meta-analysis assessing the impact of community-level sanitation access on child stunting, anemia, and diarrhea: Evidence from DHS and MICS surveys

**DOI:** 10.1371/journal.pntd.0005591

**Published:** 2017-06-08

**Authors:** David A. Larsen, Thomas Grisham, Erik Slawsky, Lutchmie Narine

**Affiliations:** Syracuse University Department of Public Health, Food Studies and Nutrition; Syracuse, NY; United States of America; Universidad Nacional Autónoma de México, MEXICO

## Abstract

**Background:**

A lack of access to sanitation is an important risk factor child health, facilitating fecal-oral transmission of pathogens including soil-transmitted helminthes and various causes of diarrheal disease. We conducted a meta-analysis of cross-sectional surveys to determine the impact that community-level sanitation access has on child health for children with and without household sanitation access.

**Methodology/Principal findings:**

Using 301 two-stage demographic health surveys and multiple indicator cluster surveys conducted between 1990 and 2015 we calculated the sanitation access in the community as the proportion of households in the sampled cluster that had household access to any type of sanitation facility. We then conducted exact matching of children based on various predictors of living in a community with high access to sanitation. Using logistic regression with the matched group as a random intercept we examined the association between the child health outcomes of stunted growth, any anemia, moderate or severe anemia, and diarrhea in the previous two weeks and the exposure of living in a community with varying degrees of community-level sanitation access. For children with household-level sanitation access, living in a community with 100% sanitation access was associated with lowered odds of stunting (adjusted odds ratio [AOR] = 0.97, 95%; confidence interval (CI) = 0.94–1.00; n = 14,153 matched groups, 1,175,167 children), any anemia (AOR = 0.73; 95% CI = 0.67–0.78; n = 5,319 matched groups, 299,033 children), moderate or severe anemia (AOR = 0.72, 95% CI = 0.68–0.77; n = 5,319 matched groups, 299,033 children) and diarrhea (AOR = 0.94; 95% CI = 0.91–0.97); n = 16,379 matched groups, 1,603,731 children) compared to living in a community with < 30% sanitation access. For children without household-level sanitation access, living in communities with 0% sanitation access was associated with higher odds of stunting (AOR = 1.04, 95% CI = 1.02–1.06; n = 14,153 matched groups, 1,175,167 children), any anemia (AOR = 1.05, 95% CI = 1.00–1.09; n = 5,319 matched groups, 299,033 children), moderate or severe anemia (AOR = 1.04, 95% CI = 1.00–1.09; n = 5,319 matched groups, 299,033 children) but not diarrhea (AOR = 1.00, 95% CI = 0.98–1.02; n = 16,379 matched groups, 1,603,731 children) compared to children without household-level sanitation access living in communities with 1–30% sanitation access.

**Conclusions/Significance:**

Community-level sanitation access is associated with improved child health outcomes independent of household-level sanitation access. The proportion of children living in communities with 100% sanitation access throughout the world is appallingly low. Ensuring sanitation access to all by 2030 will greatly improve child health.

## Author summary

A lack of access to a sanitation facility, i.e. a toilet and/or latrine, leads to numerous health challenges such as parasitic worms and environmental enteropathy. Parasitic worms are transmitted through human feces and cause multiple health complications in children including anemia and child growth stunting. Environmental enteropathy occurs with repeated and long-term inflammation of the small intestine which then reduces nutrient uptake and can cause child growth stunting, anemia and diarrhea. One-sixth of the world population has no access to any type of sanitation facility, and are therefore at higher risk of these challenges. Scientific literature on the impacts of sanitation typically examines household access to sanitation rather than community-level access to sanitation. We used national survey data to assess the impact that community-level access to sanitation has on child health, both for children with access to a sanitation facility and children without access to a sanitation facility. We found that a lack of sanitation access in the community is a significant risk factor for anemia and child growth stunting, but not for incidence of diarrhea. This risk decreases if a child has access to a sanitation facility, but even among those children with a sanitation facility poor sanitation access in the community is still a risk factor for anemia, child growth stunting and diarrhea. In addition to improving household access to adequate sanitation, community-level sanitation access needs to be addressed to improve child health. These results will add impetus to the Sustainable Development Goal to ensure sanitation access for all by 2030.

## Introduction

An estimated 1 billion people live without access to any type of sanitation facility, i.e. a toilet or latrine [1]. This lack of sanitation access fails to contain human feces, which are responsible for transmission of various diarrheal diseases as well as soil-transmitted helminthes (STH) primarily through the fecal-oral route where fecal matter is ingested via water, dirt or food [2]. Diarrheal diseases kill millions of children each year [3], and for those who survive present the problem of malnutrition and developmental delays [4]. STH cause malnutrition and stunting in addition to developmental delays [5]. Furthermore hookworm (*Necator americanus* or *Ancylostoma duodenale*) are known risk factors for anemia [6]. Infections with *Ascaris lumbricoides* (roundworm) and *Trichuris trichiura* (whipworm) may also be risk factors for anemia although the evidence is inconclusive [7].

The prevalence of anemia is high in lower-income countries, estimated at 47% of children in 2005 [8], though recent reports suggest the prevalence is decreasing [9]. Due to the importance of iron to various cellular functions including immune system functionality [10,11], iron deficiency anemia is implicated as a cause of mortality for millions of children under five years of age each year [12,13]. Beyond a cause of mortality, anemia also decreases cognitive function [14–16], and energy levels which leads to decreased productivity and economic well-being [17,18]. For subsistence farmers in lower-income countries decreased productivity can in turn lead to low crop yields and food insecurity, perpetuating a vicious cycle of malnutrition.

Through containment and disposal of human feces, individual-level access to sanitation is known to decrease both diarrheal disease and STH infection [19–23]. A previous examination of survey data 1986–2007 found decreased risk of child mortality, diarrhea and stunting for children living in households with access to improved sanitation [24]. However, limiting sanitation to a household-level risk factor while ignoring the community-effect may greatly underestimate the impact that sanitation has on human health [25]. Poor sanitation in the community leads to increased exposure to fecal matter for all in that community, a significant risk factor for environmental enteropathy and subsequent child malnutrition [26]. Indeed, in India the behavior of open defecation was associated with reductions in child growth in an ecological analysis [27], and in Cambodia community-sanitation behavior was associated with increased child growth more prominently than household-sanitation behavior [28]. Numerous community-randomized controlled trials of total sanitation campaigns have suggested that increasing access to sanitation can improve child health [28–31], while others have found little to no effect of these interventions on child health [32–34].

Herein we present a study estimating the impact of community-level access to sanitation on child health as measured through child growth, anemia, and diarrhea symptoms using survey data compiled into an individual-level meta-analysis.

## Methods

### Study design

We sought to measure the impact that living in a community with 100% sanitation access has on the outcomes of child growth stunting among children aged 12–59 months, anemia among children under 5 years of age, and diarrhea in the previous two weeks from nationally-representative surveys. To do so we pooled surveys to create an individual-level meta-analysis [35].

### Setting

We included multiple indicator cluster surveys (MICS), demographic and health surveys (DHS), malaria indicator surveys (MIS), and AIDS indicator surveys (AIS) that were nationally-representative and publicly available as of July 2016. As part of original survey protocol all data were anonymized prior to download from repositories to protect participant privacy.

### Outcomes

Anthropomorphic data are regularly collected in nationally-representative surveys. In these surveys height for age z-scores are computed for children under 5 years of age based upon World Health Organization growth reference standards. We classified children as stunted or not based upon the child’s height for age z-score being less than 2 standard deviations of the WHO growth reference standard. The outcome of stunting was available for 267 of 301 datasets. Nationally-representative surveys typically use the HemoCue system to measure hemoglobin levels for children age 5 and under and adjust these values for altitude. Depending upon the level of hemoglobin in the blood anemia is classified as none (≥12.0 g/dl), mild (10.0–11.9 g/dl), moderate (7.0–9.9 g/dl), and severe (< 7.0 g/dl). We conducted analyses with two separate anemia outcomes, children with any anemia (mild, moderate, or severe) and children with moderate to severe anemia. The anemia outcomes were available for 104 of 301 datasets. Caregivers of children under five are also asked whether their child has had any commonly occurring illnesses such as fever, diarrhea, or cough. We classified children with diarrhea as those whose caregivers reported them having diarrhea in the previous 2 weeks, and children without diarrhea as those whose caregivers reported them not having diarrhea in the previous 2 weeks. The outcome of diarrhea was available for 281 of 301 datasets.

### Primary exposure

In order to estimate the incremental effect of increasing community-level sanitation access on the outcomes of child growth stunting and anemia among children we classified children as living in households with any type of sanitation facility (unimproved or improved), or not having any access to a sanitation facility. If households reported sharing a sanitation facility with others they were classified as having any type of sanitation facility. We defined community as the survey sampling area or cluster, and calculated the proportion of households having any sanitation facility (unimproved or improved) to serve as a measure of community-level sanitation access. We excluded datasets where > 95% of children live in communities with 100% sanitation access from any further analyses.

### Bias

Children in households with sanitation facilities or in communities with high sanitation access are likely to be predisposed to less risk of stunting and anemia, independent of sanitation access. To account for this selection bias and potential confounding we used two separate methodologies. First, we stratified our analyses by children in households with any sanitation access and children in households without any sanitation access. Second we used exact matching on community-level measures to circumvent the inherent selection bias of living in communities with more access to sanitation. Using the MatchIt package [36] in R version 3.2.3 [37] we matched children on numerous community-level and other covariates. To do so, we first took the cluster mean of child-level immunization coverage (3 doses of diphtheria, pertussis and tetanus). We then took the cluster mean of household wealth quintile and household access to a water source that was not considered surface water (rivers, dams, ponds, lakes or unprotected springs). Once these cluster-level estimates were estimated we categorized estimates of cluster-level immunizations into tertiles, community-level wealth above and below the median, and community-level access to a non-surface water source above and below the median. In addition to the community-level measures we matched on household-level wealth (dichotomized into rich or poor) and mother’s education (dichotomized into completed primary or not). The exact matching was conducted in accordance with the following equation: *m*_*ijkl*_ = *β*_0_ + *βC*_*i*_ + *χH*_*j*_ + *δP*_*k*_ + *ϕS*_*l*_ where m_ijk_ is a matched group for child *i* in household *j* in cluster *k* in survey *l*, *C*_*i*_ is an estimate of the mother’s education, *H*_*j*_ is an estimate of household wealth, *P*_*k*_ is a vector of cluster characteristics and *S*_*l*_ is a survey dummy. The matching procedures and all covariates were selected a priori.

### Statistical analysis

Pooling all datasets to create an individual-level meta-analysis we first examined the relationship between the outcomes and community-level sanitation access through a Lowess smoothing figure. To account for observable non-linearity in the exposure of interest we attempted to fit a cubic spline, however the spline was unable to account for the large decrease in the odds of the outcomes when going from 99% sanitation access to 100% sanitation access. We therefore categorized community-level sanitation access at 0%, 1–30%, 31–60%, 61–99%, and 100% to both align with the knots of the cubic spline (0.6 and 0.99) and to provide an appropriate comparison group (1–30%).

Second, we calculated the unadjusted association between the exposures and outcomes of interest. For the unadjusted analysis we included the dataset and household sanitation access as covariates and adjusted the standard errors for correlated data at the survey cluster level. Finally, we used a generalized linear model with the matched group as a random intercept and a logit link to assess an adjusted association between the exposures and outcomes of interest. We included the following covariates to decrease the potential for confounding, with variable selection determined a priori: household sanitation access, urban or rural, child’s age in years, mother’s education (quantified as none, some, and completed primary or higher), household wealth quintile, insecticide treated mosquito net (ITN) coverage (no ITN in household, household owns ITN but child did not use previous night, and child used ITN previous night), child’s weight for height (no wasting, 0–2 standard deviations below reference, >2 standard deviations below reference), child has a health or immunization card (no, yes), child immunizations (none, some, or all according to WHO standards), previous birth interval (< 24 months or not), birth order (firstborn, second born, third born, or later), mother’s age of the child in 5 year increments (i.e., 15–19, 20–24, etc.), household size in terms of number of people (<6, 6–15, >15), whether the household uses an open water source (defined as a river, stream, pond, or unprotected spring), national gross domestic product retrieved from the World Bank database for the year of the survey as a continuous variable, and dataset as a dummy variable.

The general model we use to assess the relationship between the outcomes and the exposure of interest is given by the following equations:

$$\begin{array}{l} y_{ijklm}|\pi_{ijklm}\sim Binomial(1,\pi_{ijklm})\\ \log it(\pi_{ijklm})=\beta_{1}San_{j}\times \beta_{2}San_{k}+\chi C_{ijk}+\delta H_{j}+\kappa S_{l}+\zeta_{m}\\ \zeta_{m}\sim N(0,\psi ) \end{array}$$

where *π*_*ijklm*_ is a dichotomous outcome for child *i* in household *j* in cluster *k* in survey *l* in matched group *m*, *Sa*n_j_ is whether the household has access to any sanitation or not, *San*_*k*_ is the level of sanitation access in the community, *C*_*ijk*_ is a vector of child characteristics, *H*_*j*_ is a vector of household characteristics, *S*_*l*_ is a vector of survey characteristics and ζ_m_ is a random intercept for matched group *m* that is assumed to be normally distributed with a mean of zero. All analyses were conducted in Stata version 13.1.

## Results

**Fig 1:**
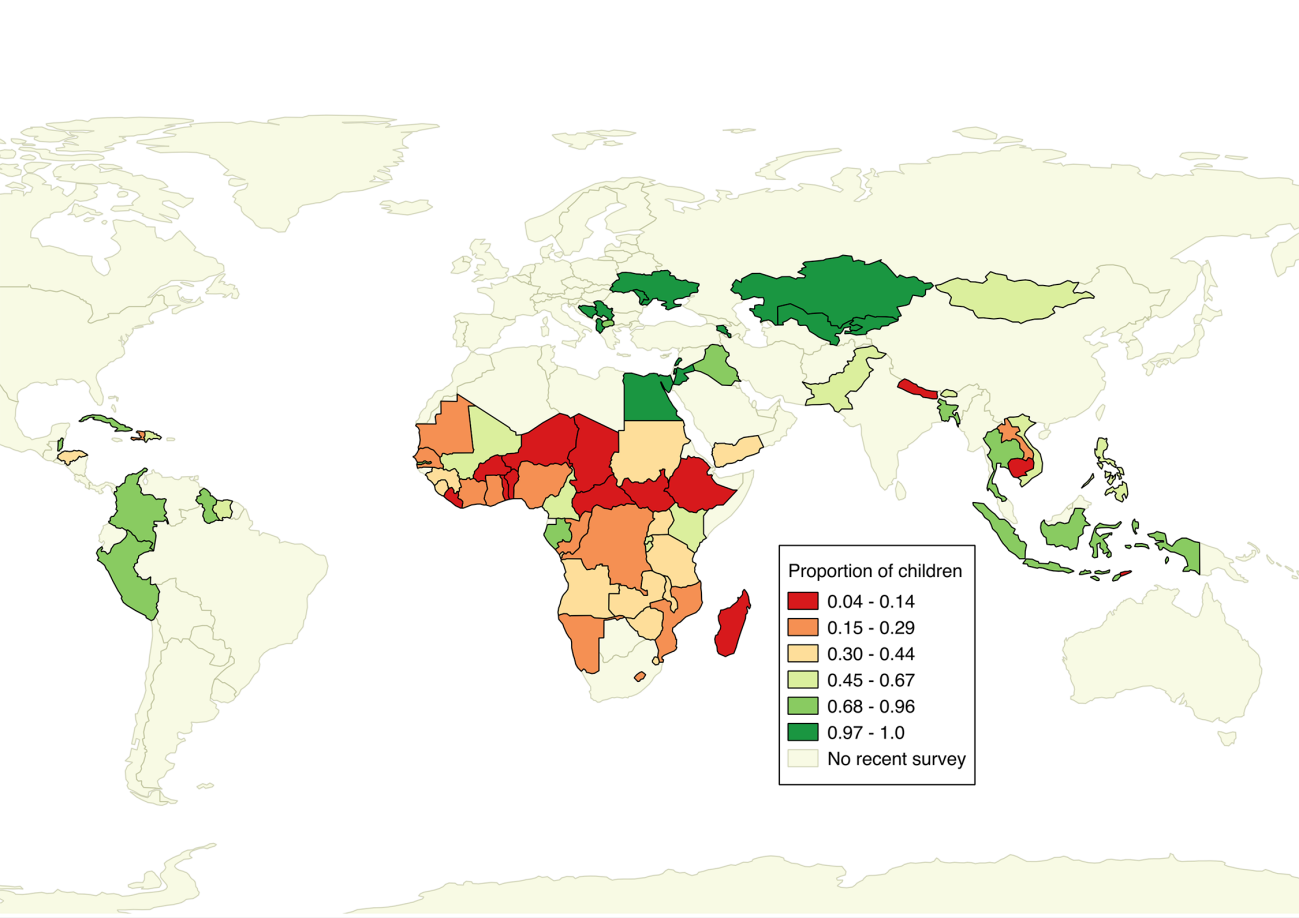
Map showing the proportion of children under 5 years of age living in communities with 100% sanitation access as measured by the most recent nationally representative survey.

We identified 301 publicly available two-stage cluster surveys from 93 separate countries beginning in 1991 and conducted as recently as 2015. Table 1 gives descriptive statistics on household and community-level access to sanitation, as well as the outcomes of stunting, anemia and diarrhea before matching. While access to sanitation has reportedly increased throughout lower-income countries, the proportion of children under 5 years of age living in communities with 100% sanitation access remains low throughout much of the world (Fig 1).

**Table 1:** Descriptive frequencies of outcomes and exposures for children in the datasets before matching.

	Household sanitation access	0% community sanitation access	1–30% community sanitation access	31–60% community sanitation access	61–99% community sanitation access	100% community sanitation access	Total
N children stunted growth (%)	No	37,050 (43%)	58,902 (40%)	38,529 (40%)	28,494 (39%)	N/A	162,975 (40%)
	Yes	N/A	10,994 (36%)	28,365 (34%)	132,380 (32%)	138,940 (21%)	310,679 (26%)
N children with any anemia (%)	No	18,498 (71%)	31,346 (68%)	14,530 (64%)	12,034 (63%)	N/A	76,408 (67%)
	Yes	N/A	5,729 (65%)	12,163 (62%)	65,533 (59%)	79,359 (47%)	162,784 (53%)
N children with moderate or severe anemia (%)	No	12,016 (46%)	19,823 (43%)	8,902 (40%)	7,258 (38%)	N/A	47,999 (42%)
	Yes	N/A	3,609 (41%)	7,376 (38%)	38,505 (35%)	40,197 (24%)	89,687 (29%)
N children with diarrhea in previous two weeks (%)	None	21,306 (18%)	35,744 (18%)	23,934 (19%)	18,294 (19%)	N/A	99,278 (18%)
	Yes	N/A	6,927 (17%)	19,699 (18%)	93,635 (17%)	120,540 (14%)	240,801 (16%)

Before matching, these 301 datasets contained anthropomorphic information for 1,592,914 children under 5 years of age, measured levels of anemia for 424,334 children under 5 years of age, and reported symptoms of diarrhea for 2,140,805 (S1 File). The matched datasets contained anthropomorphic information for 1,197,371 children from 233 datasets, measured levels of anemia for 299,560 children from 93 datasets, and reported symptoms of diarrhea for 1,616,619 children from 247 datasets. (See S1 Data for Prisma framework).

### Community-level sanitation access

**Fig 2:**
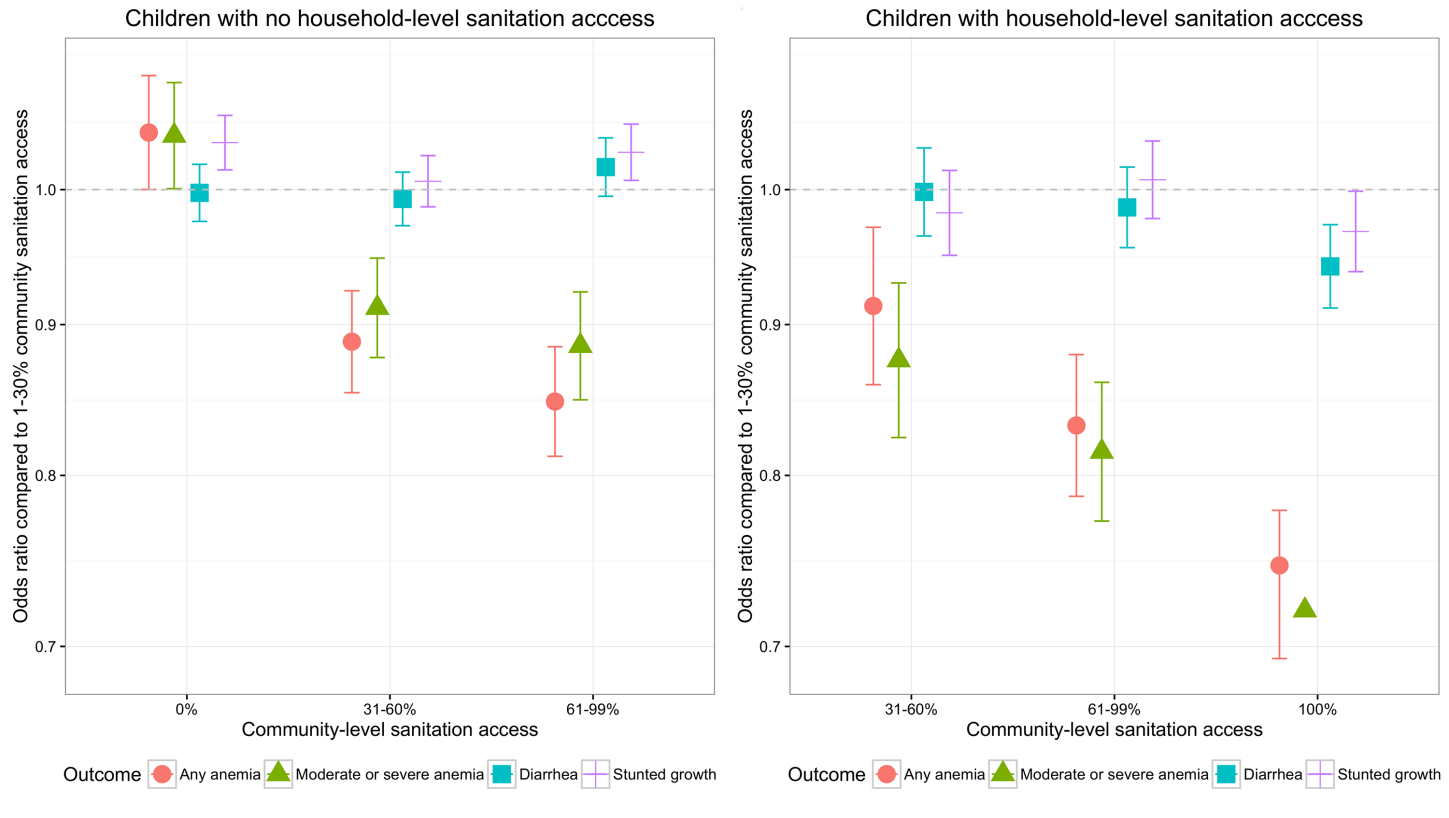
Adjusted odds ratios of the impact of increasing levels of community-level sanitation access on the outcomes of child growth stunting, any anemia, moderate or severe anemia, and reported symptoms of diarrhea in the previous two weeks for children living in households with and without sanitation access.

Among children living in households with sanitation access, living in a community with 100% sanitation access is associated with lower odds of stunting (Table 2). The lower odds of being stunted is only observed at 100% sanitation access; there was no effect of increasing community-level sanitation access for children in households with a sanitation facility located in clusters with < 100% sanitation access (Fig 2). Among children living in households without sanitation access, living in communities with zero sanitation access was associated with higher odds of stunting compared to children living in communities with 1–60% sanitation access (Table 2). Among children living in communities with high access to sanitation (60% or more of households with sanitation access) not having household-level sanitation access was associated with higher odds of stunting compared to children living in communities with 1–60% sanitation access (Fig 2; Table 2).

**Table 2:** Associations between stunted growth (< 2 standard deviations below WHO reference population) and level of sanitation access in the community for children with and without household-level access to sanitation. Unadjusted odds ratios derived from logistic regression adjusted for dataset and with robust standard errors to account for correlated data at the EA-level. Adjusted odds ratios derived from logistic regression with matched group as a random intercept. Models adjusted for urban/rural, child’s age, wealth quintile, mother’s education, child’s immunization status, child having a health or immunization card, mother’s age, household size, previous birth interval, household water source, national gross domestic product and dataset. N = 14,153 matched groups; 1,175,167 children under the age of 5.

Household sanitation access	Community-level sanitation access	Unadjusted odds ratio (95% confidence interval)	P-value	Adjusted odds ratio (95% confidence interval)	P-value
No	None	1.12 (1.09–1.16)	< 0.0001	1.04 (1.02–1.06)	0.001
No	1–30%	Reference	Reference	Reference	Reference
No	31–60%	0.94 (0.92–0.97)	< 0.0001	1.01 (0.99–1.03)	0.521
No	60–99%	0.89 (0.87–0.91)	< 0.0001	1.03 (1.01–1.05)	0.009
Yes	1–30%	Reference	Reference	Reference	Reference
Yes	31–60%	0.92 (0.89–0.96)	< 0.0001	0.98 (0.95–1.02)	0.283
Yes	60%–99%	0.86 (0.83–0.89)	< 0.0001	1.01 (0.98–1.04)	0.619
Yes	100%	0.64 (0.62–0.67)	< 0.0001	0.97 (0.94–1.00)	0.041

For the outcomes of any anemia as well as moderate or severe anemia, increasing community-level access to sanitation is associated with lower odds of anemia for children in households with sanitation access as well as children in households without sanitation access (Fig 2; any anemia Table 3; moderate or severe anemia Table 4). Increasing protection for all children occurred with increasing community-level sanitation access.

**Table 3:** Associations between the outcomes of any anemia (< 12.0 mg hemoglobin / dl of blood) and level of sanitation access in the community for children with and without household-level access to sanitation. Unadjusted odds ratios derived from logistic regression adjusted for dataset and with robust standard errors to account for correlated data at the EA-level. Adjusted odds ratios derived from logistic regression with matched group as a random intercept. Models adjusted for urban/rural, child’s age, wealth quintile, child’s weight for height (wasting), insecticide treated net ownership and use, mother’s education, child’s immunization status, child having a health or immunizations card, previous birth interval, mother’s age, household size, household water source, national gross domestic product and dataset. N = 5,319 matched groups; 299,033 children under the age of 5.

Household sanitation access	Community-level sanitation access	Unadjusted odds ratio (95% confidence interval)	P-value	Adjusted odds ratio (95% confidence interval)	P-value
No	None	1.10 (1.04–1.17)	0.001	1.05 (1.00–1.09)	0.050
No	1–30%	Reference	Reference	Reference	Reference
No	31–60%	0.87 (0.83–0.92)	< 0.0001	0.89 (0.85–0.92)	< 0.0001
No	60–99%	0.82 (0.78–0.86)	< 0.0001	0.85 (0.81–0.88)	< 0.0001
Yes	1–33%	Reference	Reference	Reference	Reference
Yes	31–60%	0.89 (0.84–0.96)	0.001	0.91 (0.86–0.97)	0.004
Yes	61%–99%	0.80 (0.75–0.84)	< 0.0001	0.83 (0.79–0.88)	< 0.0001
Yes	100%	0.65 (0.61–0.69)	< 0.0001	0.73 (0.69–0.78)	< 0.0001

**Table 4:** Associations between moderate or severe anemia (< 10.0 mg hemoglobin / dl of blood) and level of sanitation access in the community for children with and without household-level access to sanitation. Unadjusted odds ratios derived from logistic regression adjusted for dataset and with robust standard errors to account for correlated data at the EA-level. Adjusted odds ratios derived from logistic regression with matched group as a random intercept. Models adjusted for urban/rural, child’s age, wealth quintile, child’s weight for height (wasting), insecticide treated net ownership and use, mother’s education, child’s immunization status, child having a health or immunizations card, previous birth interval, mother’s age, household size, household water source, national gross domestic product and dataset. N = 5,319 matched groups; 299,033 children under the age of 5.

Household sanitation access	Community-level sanitation access	Unadjusted odds ratio (95% confidence interval)	P-value	Adjusted odds ratio (95% confidence interval)	P-value
No	None	1.10 (1.04–1.16)	<0.0001	1.04 (1.00–1.09)	0.046
No	1–30%	Reference	Reference	Reference	Reference
No	31–60%	0.89 (0.85–0.93)	<0.0001	0.91 (0.88–0.95)	< 0.0001
No	61–99%	0.84 (0.80–0.88)	<0.0001	0.89 (0.85–0.92)	< 0.0001
Yes	1–30%	Reference	Reference	Reference	Reference
Yes	31–60%	0.86 (0.80–0.91)	< 0.0001	0.88 (0.82–0.93)	< 0.0001
Yes	61%–99%	0.77 (0.73–0.81)	< 0.0001	0.81 (0.77–0.86)	< 0.0001
Yes	100%	0.62 (0.58–0.65)	< 0.0001	0.72 (0.68–0.76)	< 0.0001

For the outcome of diarrhea symptoms in the previous two weeks, increasing community-level access to sanitation is not associated with lower odds of diarrhea for children in households without access to sanitation (Fig 2, Table 5). For children in households with access to sanitation living in a community with 100% sanitation access was associated with a lower odds of diarrhea (Fig 2, Table 5).

**Table 5:** Associations between reported symptoms of diarrhea in the previous two weeks and level of sanitation access in the community for children with and without household-level access to sanitation. Unadjusted odds ratios derived from logistic regression adjusted for dataset and with robust standard errors to account for correlated data at the EA-level. Adjusted odds ratios derived from logistic regression with matched group as a random intercept. Models adjusted for urban/rural, child’s age, wealth quintile, mother’s education, child’s immunization status, child having a health or immunization card, mother’s age, household size, previous birth interval, household water source, national gross domestic product and dataset. N = 16,379 matched groups; 1,603,731 children under the age of 5.

Household sanitation access	Community-level sanitation access	Unadjusted odds ratio (95% confidence interval)	P-value	Adjusted odds ratio (95% confidence interval)	P-value
No	None	1.00 (0.97–1.03)	0.913	1.00 (0.98–1.02)	0.825
No	1–30%	Reference	Reference	Reference	Reference
No	31–60%	0.99 (0.96–1.01)	0.311	0.99 (0.97–1.01)	0.495
No	61–99%	0.99 (0.97–1.02)	0.617	1.02 (0.99–1.04)	0.130
Yes	1–30%	Reference	Reference	Reference	Reference
Yes	31–60%	0.98 (0.95–1.03)	0.455	1.00 (0.96–1.03)	0.919
Yes	61%–99%	0.94 (0.90–0.97)	< 0.0001	0.99 (0.96–1.02)	0.388
Yes	100%	0.83 (0.80–0.86)	< 0.0001	0.94 (0.91–0.97)	< 0.0001

### Household-level sanitation access

Children living in houses with access to a sanitation facility was associated with lower odds of stunting and any anemia at all levels of community-access to sanitation compared to children in houses with no access to a sanitation facility (Table 6). Living in a house with access to a sanitation facility was associated with lower odds of the outcomes of moderate or severe anemia and diarrhea compared to living in a house with no access to a sanitation facility only when community-sanitation access was higher.

**Table 6:** Association between household-level access to a sanitation facility and the outomes. All odds ratios compare having a sanitation facility to not. Models are the same as those described in previous tables.

Community-level sanitation access	OR for stunting (95% CI)	OR for any anemia (95% CI)	OR for moderate or severe anemia (95% CI)	OR for diarrhea (95% CI)
1–30%	0.94 (0.91–0.97)	0.91 (0.86–0.97)	0.98 (0.93–1.04)	0.97 (0.94–1.01)
31–60%	0.91 (0.89–0.93)	0.94 (0.90–0.98)	0.94 (0.90–0.99)	0.98 (0.96–1.00)
61–99%	0.92 (0.90–0.93)	0.90 (0.86–0.93)	0.90 (0.97–0.94)	0.94 (0.93–0.96)

## Discussion

We found that community-level access to sanitation is associated with lower odds of stunting and anemia for children independent of household-level sanitation access, and lower odds of diarrhea for children in houses with a sanitation facility. For children with sanitation access our analyses suggest that further gains in reducing the risk of stunting, anemia and diarrhea can be made as their communities move toward universal sanitation access. For children without household-level sanitation access our analyses suggest that community-level sanitation in addition to household-level sanitation is an important factor in child health.

Unexpectedly for children without individual-level access to sanitation, living in a community with higher access to sanitation (60–99%) was not beneficial compared to living in a community with no access to sanitation in terms of both stunting and diarrhea. (It was beneficial for the outcome of anemia). We suspect that lacking a sanitation facility when the majority of neighbors have one is an indicator of vulnerability and for an outcome such as stunting with a multi-factorial causal etiology the vulnerability may represent a risk factor. In contrast for the outcome of anemia a significant benefit was observed for this particular population.

Diarrhea in the previous two weeks was not associated with community-level access to sanitation, except for those children living in communities with 100% sanitation access. Also household-level access to sanitation was only associated with lower odds of diarrhea when community-level sanitation exceeded 60%. These findings that found improved sanitation at the household level to be associated with lowered risk of diarrhea [24]. Unmeasured confounding is a primary threat to these types of analyses, and the lack of impact may be due to unmeasured risk factors. Furthermore, there is great uncertainty around the validity of self-reported diarrhea in surveys [38], and the subsequent misclassification error may lead to an underestimation of the impact of sanitation on diarrhea. Decreased fecal matter in the environment is likely to decrease circulation of diarrhea-causing agents, however there was no way to account for handwashing behavior in this analysis which is suggested to drive the relationship between diarrheal disease and sanitation [39,40].

The association between higher community-level sanitation access and the outcomes of anemia and stunting (at lower levels of community-level access) are consistent with the theory that environmental enteropathy is a significant risk factor for child malnutrition and health [26]. A recent modeling analysis and literature review suggests that community-level sanitation acts through a type of “herd-immunity” mechanism [41], and an observational study demonstrated the protective nature of herd-immunity from sanitation in rural Ecuador [42]. These analyses confirm that a lack of sanitation at the community level poses a risk to members of that community, independent of household sanitation access and that the greatest gains occur as communities achieve universal access to sanitation.

Our findings are in line with the scientific understanding of how fecal-oral transmission of various pathogens impact child health [26,41]. The measurement of sanitation access at the level of primary sampling unit of nationally-representative surveys is an innovation that improves upon previous analyses of survey data that only measure sanitation access at the district level [27], or consider sanitation as a household-level risk factor [24]. Still, these findings should be treated cautiously for a number of reasons. First we greatly simplified sanitation access as having a sanitation facility or not. The sanitation ladder is much more nuanced [43], with the greatest benefits to health coming from improving sanitation beyond a simple pit latrine. The simplification of sanitation access to having a facility or not allowed for its measurement at community level. Second, survey data are subject to error including recall and information error. Both outcomes included in this analysis were measured by survey personnel, and are not likely to be associated with the exposure of interest. However responses in survey questions about sanitation access may have suffered from social-desirability bias. Finally the use of the primary sampling unit as the community is not a perfect measure of community, given that primary sampling units may comprise various villages. Given the comprehensive nature of the datasets used and the random sampling of children selected we do not anticipate any publication or reporting bias to threaten the validity of these results.

These results suggest that the greatest gains in health from sanitation are made when communities achieve universal access to sanitation. Until access to sanitation is universal within a population, even those with access carry risk derived from those without access to sanitation. Access to sanitation was included in the Millennium Development Goals as target 7.C, with the goal of reducing by half the population without access to safe drinking water and basic sanitation. Progress was minimal; the target was missed by nearly 1 billion people [1]. These data show that poor community-level sanitation access is a significant risk factor for child growth stunting and anemia, both for children living in households with access to sanitation and for children living in households without. The number of children living in communities where any households lack sanitation access is alarmingly high throughout the world, and efforts must be made to achieve the Sustainable Development Goal of eliminating open defecation by 2030.

## Supporting information

S1 File Supplemental file one contains the PRISMA framework outlining the process for including datasets in the analysis. (DOC)

S1 Data Contains a table of unadjusted outcomes and exposures by dataset. (XLSX)
