# Supplementary material for: An individual-level meta-analysis assessing the impact of community-level sanitation access on child stunting, anemia, and diarrhea: Evidence from DHS and MICS surveys
Source: PLoS Negl Trop Dis. 2017 Jun 8;11(6):e0005591. doi: 10.1371/journal.pntd.0005591 (PMC5464528; doi:10.1371/journal.pntd.0005591)
Supplement: S1 File — (DOC) [file pntd.0005591.s001.doc]

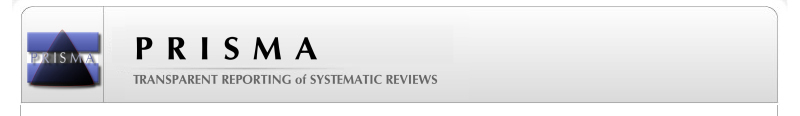
**PRISMA 2009 Flow Diagram**

**Screening**

**Included**

**Eligibility**

**Identification**

2-stage cluster surveys 1990-2016 available from DHS program (n = 295)

2-stage cluster surveys 1990-2016 available from UNICEF (n = 168)

Non-duplicate datasets with sanitation and child information (n = 301)
(n = )

Datasets with stunting outcome (n = 267)

Datasets with child anemia outcome (n = 104)

Datasets excluded because >95% children live open defecation free (n = 28)

Datasets excluded because >95% children live open defecation free (n = 8)

Datasets available before matching (n = 239)

Datasets included after matching in stunting analysis (n = 232)

Datasets excluded
(n = 165)

Datasets available before matching (n = 96)

Datasets included after matching in anemia analysis (n = 93)

Datasets with diarrhea outcome (n = 281)

Datasets excluded because >95% children live open defecation free (n = 26)

Datasets available before matching (n = 255)

Datasets included after matching in diarrhea analysis (n = 247)
